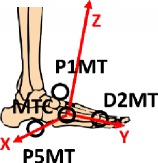# Two-Segment Foot Model for the Biomechanical Analysis of Squat

**DOI:** 10.1155/2017/9652948

**Published:** 2017-08-06

**Authors:** E. Panero, L. Gastaldi, W. Rapp

**Affiliations:** ^1^Department of Mechanical and Aerospace Engineering, Politecnico di Torino, Turin, Italy; ^2^Department of Sport and Sport Science, University of Freiburg, Freiburg, Germany

## Abstract

Squat exercise is acquiring interest in many fields, due to its benefits in improving health and its biomechanical similarities to a wide range of sport motions and the recruitment of many body segments in a single maneuver. Several researches had examined considerable biomechanical aspects of lower limbs during squat, but not without limitations. The main goal of this study focuses on the analysis of the foot contribution during a partial body weight squat, using a two-segment foot model that considers separately the forefoot and the hindfoot. The forefoot and hindfoot are articulated by the midtarsal joint. Five subjects performed a series of three trials, and results were averaged. Joint kinematics and dynamics were obtained using motion capture system, two force plates closed together, and inverse dynamics techniques. The midtarsal joint reached a dorsiflexion peak of 4°. Different strategies between subjects revealed 4° supination and 2.5° pronation of the forefoot. Vertical GRF showed 20% of body weight concentrated on the forefoot and 30% on the hindfoot. The percentages varied during motion, with a peak of 40% on the hindfoot and correspondently 10% on the forefoot, while the traditional model depicted the unique constant 50% value. Ankle peak of plantarflexion moment, power absorption, and power generation was consistent with values estimated by the one-segment model, without statistical significance.

## 1. Introduction

The squat is one of the most common total body and strength exercises that permits to increase the power, the balance, and the endurance of the trunk [1] and lower limbs [2]. Due to its biomechanical and neuromuscular similarities to a wide range of gesture performed in different sports and its ability to recruit multiple muscle groups in a single maneuver, the squatting movement is popular in several application fields. Squat is included as a core exercise in many sports routines designed to augment athletic performance, as it is widely regarded as a supreme test of lower body strength [3]. The squat is considered a crucial element in the weightlifting and powerlifting sports, but it is also linked to sprinting and vertical jump ability in the cross-country skiing [4–6], snowboarding [7], and ski jumping [8]. Indeed, squat analysis is becoming fundamental to check power and resistance of the lower limbs in athletic subjects. Benefits are not limited to the sports sphere, but the squatting movement has closed correlation to many activities of daily living, such as sitting, standing, or picking up objects. It is considered one of the best exercises to improve health and quality of life, as to test the preservation of functional independence in aged people [9]. Its popularity is increasing in clinical settings as in therapeutic and rehabilitation treatment of ligament lesions [10, 11], patellofemoral dysfunctions [12], total joint replacement [13], and ankle instability [14, 15].

The squat appears to be a basic motion, but it is one of the most complex movements to learn and it should not be performed with external resistances without mastering the body weight movement pattern. Previous analyses determined that an appropriate squat maneuver can only be performed with optimal posture, joint alignment, coordination, and absence of pain [16]. This functional and multijoint exercise has received considerable biomechanical evaluation. Several studies had examined the kinematics [17, 18], dynamics [19, 20], muscle recruitment patterns [21] at hip, and knee joints [22, 23] for different squat strategies and resistance loads [24].

Despite the importance of the topic and the numerous investigations, most kinematic and dynamic analyses only deal with the motion in the sagittal plane, neglecting the other directions. Moreover, most cases focus on the hip and knee behavior. Few researches concentrate on the ankle role because of the small value of forces and net moments [15–17] and on the body weight distribution on the foot [25, 26].

Although the foot presents a complex anatomy and it plays an essential role in numerous motions, usually it is biomechanically modeled as a single rigid segment, without considering internal foot joints. To overcome this simplification and to increase the understanding of foot functions, some multisegment kinematic foot models are developed and validated [27–32]. Nevertheless, there are only a few studies using the multisegment foot model to evaluate dynamics [33–35] and to study foot-floor contact during human motion [36]. In addition, all of them were validated in gait analysis.

The principal aim of this study deals with the use of a two-segment model for the analysis of kinematic and dynamic foot variables during a partial body weight squat. The model, developed and validated in gait analysis [35], was used for the analysis of squat performed by five healthy volunteers. The model allows to separate the forefoot and the hindfoot and to analyze the midtarsal joint biomechanics. The strategies of foot position and the different resultant forces in the two foot subareas were highlighted. Results were also evaluated at the ankle joint and then compared with the one obtained with the one-segment model, hypothesizing the absence of statistical differences in peaks of plantarflexion moment, power absorption during descending phase, and power generation in ascending phase.

## 2. Materials and Methods

### 2.1. Model

**Figure 1:**
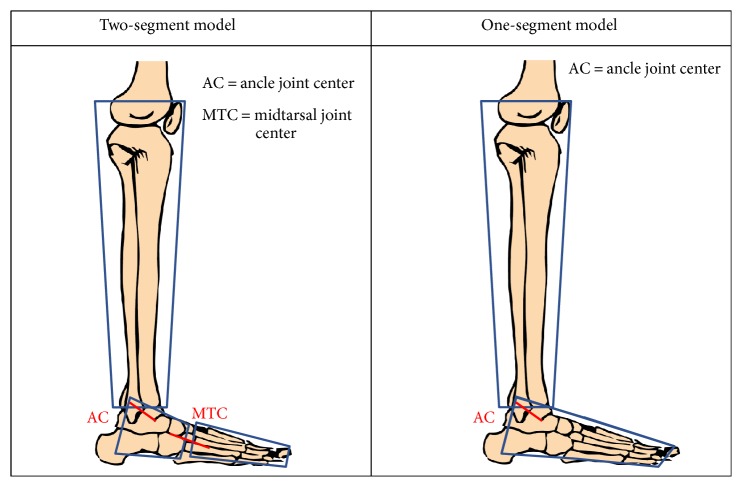
Geometrical schematization of the two-segment model and the one-segment model developed.

The two-segment model focuses on the idea to better describe the complex anatomy of foot, its internal joints, and its degrees of freedom. The model was designed for biomechanical analysis, considering both kinematic and dynamic variables. Due to platform limits on estimating one total resultant as external force, only two subareas could be considered and hence a two-segment model was developed. The application of the model and the placement of the two force plates side by side give the opportunity to simultaneously register data from the two subareas during the same trial, without mathematical assumptions or correlation. The reliability of force measures and the absence of boundary effects on plates were checked with preexperiments. Some starting tests were conducted to define which foot subareas could be identified. The division of the forefoot from the hindfoot revealed to be the most appropriate. As a consequence, the midtarsal joint was identified. At the same time, a one-segment model was assumed combining the forefoot and the hindfoot in a unique segment for the comparison of ankle joint results. The development of the model has been described in a previous study [35]. In Figure 1, a geometrical schematization of the main design of the two models is reported.

#### 2.1.1. Marker Protocol

The marker placement protocol was similar to the Plug in Gait protocol [37], with additional markers. One maker was placed on the medial femoral epicondyle for the knee joint assessment, one on the metatarsal, and one on the calcaneus bones for different foot subareas identification.  Table 1 summarizes markers and virtual landmarks with their anatomical position. A picture of all markers posed on the lower limb, and joint location is also reported. Markers used for static trial are not labeled.

#### 2.1.2. Joint Centers

Ankle joint center complex AC, separating the hindfoot from the shank, and midtarsal joint center MTC, separating the hind from the forefoot, were identified. The midpoint between femoral epicondyles was selected as the knee center KC, and it was used only for shank geometry description.

The AC was consistently modeled as the midpoint of the medial and lateral malleoli [38]. Articulations between the calcaneus and cuboid and between the talus and navicular were collectively combined in the midtarsal joint, which represents the motor and functional center of the midfoot [39]. Due to anatomical characteristics and limitations, there are several methods reported in the literature to establish its position [40]. At the beginning, as suggested by Bruening et al. [34], the midpoint between the navicular and cuboid was considered. However, this interpretation was discarded because of the difficulty in palpation of the cuboid prominence and the lack of its repeatability. Instead, the MTC was defined as the midpoint between the most medial and lateral metatarsal bases. In this way, also the tarsometatarsal joint was considered, that is an important biomechanical feature of the midfoot.

#### 2.1.3. Reference Frames

The global coordinate system (GCS) was defined during the stereophotogrammetric system calibration. The posterior right corner of the first force plate was considered as the origin. The anterior posterior *x*-axis is positive along the motion direction, and the *z*-axis is vertical and positive upwards. The Shriners Hospital foot model [41] was considered for the definition of local reference frame, and a detailed description of all segment reference frames is shown in Table 2.

#### 2.1.4. Segment Geometry

According to the Hanavan model [42] and its mathematical representation of the human body as a kinematic chain of linkages, all segments were modeled as a frustum of a circular cone with a uniform density. Referring to a static initial acquisition, the different geometric parameters of the segments were defined as the following:

- Shank: the distal diameter corresponds to the distance between femoral epicondyles, the proximal diameter corresponds to the distance between malleoli, the height of the cone corresponds to the distance between KC and AC.
- Foot: the distal diameter corresponds to the distance between malleoli, the proximal diameter corresponds to the *z*-coordinate of HLX in GCS, the length of the foot is measured directly on the subject before the test.
- Hindfoot: the distal diameter corresponds to the distance between malleoli, the proximal diameter corresponds to the distance between P1MT and P5MT, while the height of the cone corresponds to the difference between the *x*-coordinate of C1 and MTC in the GCS.
- Forefoot: the distal diameter corresponds to the distance between P1MT and P5MT, the proximal diameter corresponds to the *z*-coordinate of HLX in GCS, while the height of the cone corresponds to the *x*-coordinate difference between HLX and MTC in the GCS.

The masses of the shank and the foot were taken from Dempster [43] and were, respectively, 4.65% and 1.45% of the total body mass. Then, the foot mass was partitioned among the foot segments corresponding to their volumes.

### 2.2. Subjects

Three female and two male young and healthy volunteers participated to the experiment (height 1,74 ± 0,11 m, mass 69,8 ± 14,11 kg, and age 22,6 ± 2,07 y). None of them presented any physical impairment, nor any inadequacies to the test. Before the data registration, all subjects had been informed about the principal aim of the study and had been instructed concerning the motion. All subjects were athletes from Sports and Sports Science University; for this reason, it was easier to explain how to reproduce the movement and to avoid misunderstandings. After the static trial, each subject performed a series of 3 partial body weight squats.

### 2.3. Movement Description

**Figure 2:**
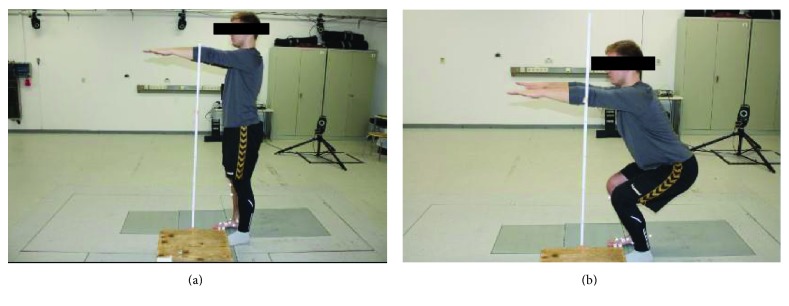
Squat motion. (a) Starting upright position with arms extended in front of the body at 90°. (b) Brief pause when the subject reached the partial squatting pose, after the downward phase and before the rising.

The participants were instructed in the performance of partial body weight squat. After the static trial, they executed a downward squat exercise starting from an upright position. Participants were asked to maintain the upper arm extended to 90° with respect to the shoulder joint (Figure 2(a)).

The squat was performed by each subject with the right foot leaned on the two force plates closed together and the left foot placed on the floor. The right foot had to be placed across the two plates in correspondence of the midfoot, with the clear identification and separation of the hind and forefoot. The subjects were previously instructed to maintain the feet in that position during squatting.

A bar with a graduate scale was laterally placed next to the subject. Two marks of colored plastic were fixed on the bar. The highest corresponded to the arm position in a standing posture; the second one was marked 40 cm lower, to give to the subject the reference for the depth of the squat. The subjects performed the series of 3 squats at a self-selected pace. At the initial upright position, the subjects were required to squat after hearing a verbal command, to stop when arms arrived at the second mark (Figure 2(b)), to stay on the target for a short time, and to return to the initial position.

### 2.4. Equipment

Principal instruments used for the data collection can be summed up as follows:

- 12 cameras Vicon MX System and Vicon Nexus Software version 1.7.1 collecting at 200 Hz;
- 12 mm diameter spherical retroreflective markers;
- AMTI force plates type BP-600900 and type OR6-7 closed together collecting at 1000 Hz;
- Laptop with Mokka 0.6 Motion Analyzer for the reconstruction of foot model, Matlab® 2015b as data processing software for all biomechanical calculations, and SPSS software v22 for statistical analysis.

### 2.5. Data and Statistical Analysis

Before the quantitative elaboration, the registered motions were qualitatively evaluated. Only the trials without lack of signal registration or marker occlusion were considered good and then selected. For each subject, two different squat trials were considered for the kinematics, while only one trial for the dynamics. Marker positions were imported and converted with Mokka for the labelling and for the model construction. Afterwards, values were elaborated using custom Matlab routines. Marker coordinate data were filtered with a low pass 4-order Butterworth filter with a cutoff frequency at 6 Hz, while force plate data were filtered with a low pass 2-order Butterworth filter with a cutoff frequency at 25 Hz.

For all the kinematic and dynamic variables, the mean value and the standard deviation (SD) were rated on all subjects. For the qualitative examination, time was normalized with respect to the squat cycle so that 100% of the motion occurred when the subjects returned to the initial position. Moreover, the dynamic values were referred to subject body mass. 3D joint angles were calculated using the Euler/Cardan rotation sequence XZY. The components of ground reaction forces (GRFs), normalized to the body weight percentage (% BW), were compared between the one-segment and two-segment models to highlight differences about the weight distribution in foot subareas. To simultaneously obtain results from the two-segment and one-segment model, the two plates were considered separately or were combined into a unique virtual force plate. The inverse dynamics was adopted for the evaluation of intersegmental forces, net joint moments, and powers. Joint power was estimated as the dot product of joint moment and joint angular velocity vector. The peak power of absorption was estimated as the maximum during descending phase, while the peak generation power as the maximum along ascending phase.

The Mann–Whitney test was used to assess any statistical differences between the two models for the peak of plantarflexion ankle moments, peak of power absorption, and peak of power generation. All statistical procedures were conducted using SPSS software version 22, and a statistical significance was considered for *p* value < 0.05.

## 3. Results

### 3.1. Joint Angles

**Figure 3:**
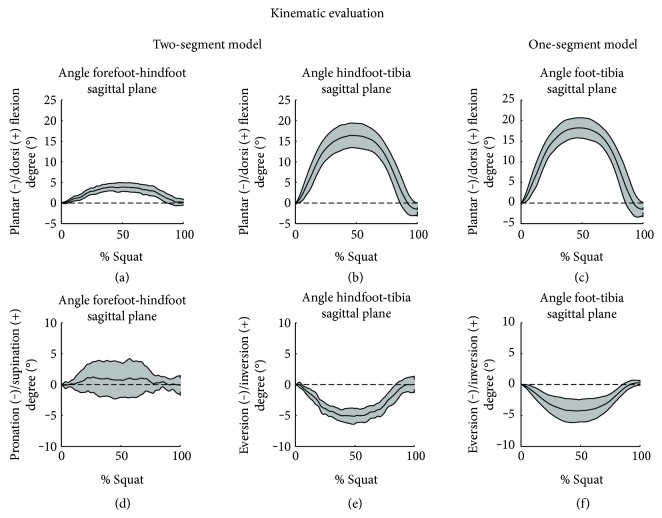
Joint angles estimated with the two-segment model (the forefoot relative to the hindfoot—hindfoot relative to the tibia) and the one-segment model (foot relative to tibia). In each graph, the average curve is reported (black line). The range of motion between the subjects is limited by the SD curves (grey area).

Kinematics was evaluated, using both the one-segment and two-segment models, in sagittal, coronal, and transverse planes. The two-segment model permitted to highlight the angle excursion at the midtarsal joint, considering the relative motion between the forefoot and hindfoot. The average joint angle patterns and the correspondent standard deviation distributions are reported in Figure 3. Each motion was normalized in agreement to the upright position assumed at the start. This strategy permitted to avoid the discrepancies caused by different starting pose among the subjects.

In the sagittal plane, a dorsiflexion was registered both at the midtarsal and at the ankle joints. The dorsiflexion peak reached 4° at the midtarsal joint, while the ankle dorsiflexion peak resulted in 16.5° if calculated with the two-segment model and 18° with the one-segment model (Figures 3(a), 3(b), and 3(c)).

In the coronal plane, the angle at the midtarsal joint highlighted a small supination of the forefoot, with a 1° peak. Considering the SD, a different strategy between subjects is notable. Indeed, some of them had placed the forefoot with a supination of 4°; while in other cases, it resulted in 2.5° pronated (Figure 3(d)). At the ankle joint, the eversion registered a peak of 5° with the two-segment model (Figure 3(e)) and 4.2° with the traditional one (Figure 3(f)).

Along the transverse plane, the midtarsal joint is closed to a neutral position, with a small oscillation between 1° of abduction and 1° of adduction. At the ankle joint, both the two models registered an external rotation of 2°. Graphs of transverse plane are not reported because of the limited contributions.

### 3.2. Ground Reaction Forces (GRF)

**Figure 4:**
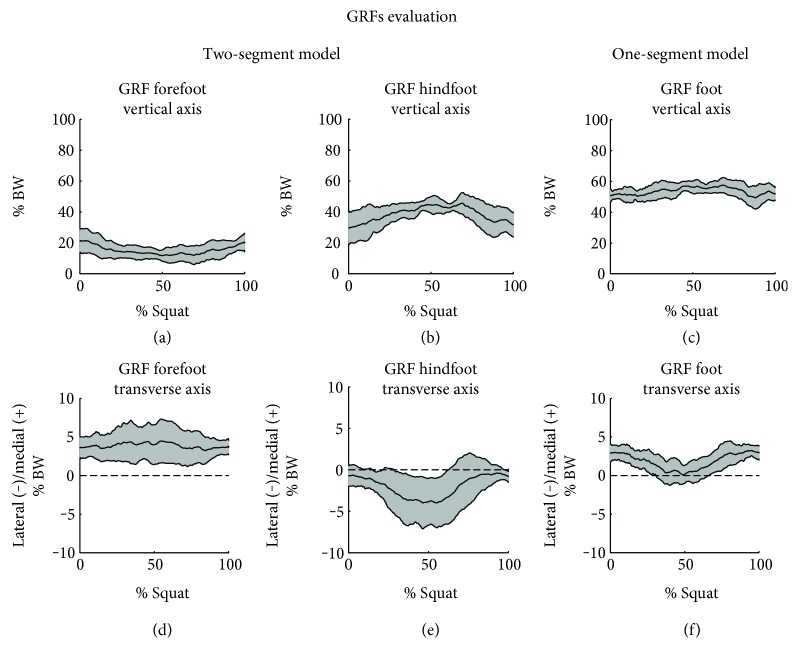
GRFs estimated with the two-segment and the one-segment models. In each graph, the average curve is reported (black line). The range of motion between the subjects is limited by the SD curves (grey area).

Before dynamic analysis, the ground reaction forces registered by the two force plates were analyzed and combined to obtain the resultant ground reaction force for the one-segment model. The force components along the vertical and transverse axes highlighted the different roles of foot subareas and the weight distribution strategy. The vertical components (Figures 4(a), 4(b), and 4(c)), as the total resultants, in the two-segment model demonstrate that 20% of BW was distributed on the forefoot and 30% on the hindfoot. During the squat cycle, the value increased on the hindfoot with a peak of 40% and decreased on the forefoot with a minimum of 10%. In the one-segment model, the vertical component (and the total resultant) showed a constant value of 50% during all the trials. In the two-segment model, the mediolateral components (Figures 4(d) and 4(e)) underline a medial contribution of the forefoot and a lateral contribution of the hindfoot that reached the peak of 5% BW. The one-segment model resumed the foot role as a lateral position (Figure 4(f)). Along the sagittal axis, the force components registered a slight contribution cause by the absence of foot translation.

### 3.3. Intersegmental Forces

**Figure 5:**
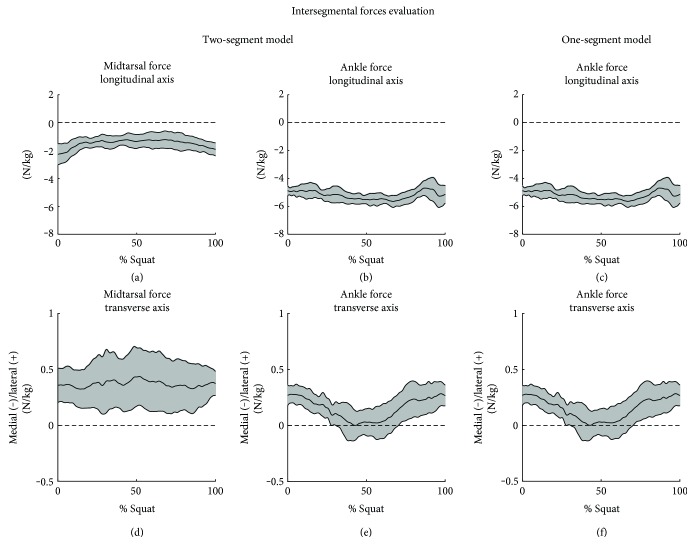
Intersegmental forces estimated with the two-segment and the one-segment models. The average curve is reported (black line). The range of motion between the subjects is limited by the SD curves (grey area).

The two-segment model permitted to evaluate not only the intersegmental force at the ankle joint, but also the force at the midtarsal joint. During the squat performance, at the midtarsal joint, the values remained approximately unvaried along the three directions, with a major contribution of −1.5 N/kg along the longitudinal axis (Figure 5(a)) and a lateral position along the transverse axis (Figure 5(d)). Results at the ankle joint were similar between the models, with a major contribution of −5 N/kg along the longitudinal axis (Figures 5(b) and 5(c)) and a medial waveform along the transverse axis (Figures 5(e) and 5(f)). Values along the sagittal axis were negligible, both for the ankle and the midtarsal joints.

### 3.4. Net Joint Moments and Powers

**Figure 6:**
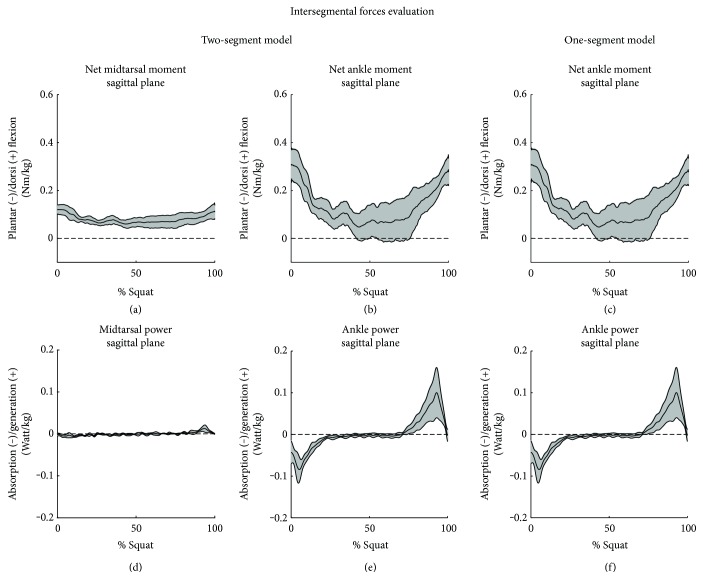
Net joint moments and powers estimated with the two-segment and the one-segment models. In each graph, the average curve is reported (black line). The range of motion between the subjects is limited by the standard deviation (grey area).

Net joint moments and powers were considered only in the sagittal plane, while other components were not reported because their contributions were slight. Results at the ankle joint were similar between the two models. The ankle net moment registered a plantarflexion waveform during the motion and reached a difference peak of −0.15 Nm/kg with respect to the starting value (Figures 6(b) and 6(c)). The midtarsal net moment contribution revealed a plantarflexion waveform with a difference peak of −0.06 Nm/kg with respect to the starting value (Figure 6(a)).

The ankle joint powers calculated with the two different models revealed similar values and waves. From the graph, the different squat phases can be identified (Figures 6(e) and 6(f)). The average peak of power absorption and power generation reached the 0.09 Watt/kg value. The joint powers developed at the midtarsal joint resulted negligible for the major of the motion, but, at the end of the ascending phase, it was possible to underline a peak of power generation of 0.01 Watt/kg (Figure 6(d)).

The Mann–Whitney test showed the absence of statistical significance between peak value of plantarflexion moments, power absorption, and power generation estimated with the two foot models (resp., *p* value = 0.35, *p* value = 0.75, and *p* value = 0.9).

## 4. Discussion

The squat is a multijoint popular exercise that is becoming fundamental for the performance and strength analysis of lower body parts in several applications, from sports and daily motion characterization to clinics and rehabilitation. Considering the complexity of the exercise and the numerous variables affected by the performance, the knowledge and investigation of squat biomechanics is of great importance for achieving awareness of the correct position as to reduce the possibility of injury. Without the complete control of body weight version, it is not recommended to add resistances or to perform variations.

The innovative scope of these experiments proposes the approach of a two-segment model for the biomechanical analysis of foot joints during the execution of a partial body weight squat. Despite its limited contribution in value of intersegmental forces and net joint moments compared with knee and hip joints, the ankle stability and the foot position play a central role for a correct and safe squat practice. The application of the two-segment model, developed and tested in a previous study [35], permits to divide the forefoot from the hindfoot, to examine internal relations, and to partly compensate the restraints provided by simplifying the foot as a one rigid segment.

As already argued by Escamilla et al. [44], a 3D kinematics was preferred to the 2D analysis because of its greater accuracy. Angles at the midtarsal and ankle joints were estimated. For the latter, a comparison with the one-segment model, pointing out a strong similarity and a considerable accuracy, was conducted. With the two-segment model, the relative motion of the forefoot with respect to the hindfoot was underlined. The dorsiflexion contribution during the descending phase shows a relative lifting of the forefoot that is confirmed by the decrease of vertical body distribution from 20% to 10% of the BW on the forefoot and the increase from 30% to 40% on the hindfoot. The analysis of the coronal plane highlighted the pronosupination of the forefoot. Zeller et al. [45] had studied differences in kinematics between men and women during the single-legged squat, and women demonstrated more ankle dorsiflexion and pronation. They associated these factors with a decreased ability of the women to maintain a varus knee position during the squat as compared with the men. Lee et al. [17] had compared kinematic angles at the hip, knee, and ankle joints in the sagittal plane during squat performed by persons with normal and pronated feet. Their experiment revealed a significant increase of ankle dorsiflexion due to the different strategies utilized by the pronated foot group. In the current case, all subjects were healthy and did not present any foot dysfunction; for this reason, the ankle dorsiflexion resulted similar. Nevertheless, also the normal subjects adopted different strategies positioning the forefoot as pronated (2.5°) or supinated (4°).

Concerning the registration of external forces, in a previous work, Kellis et al. [25] examined the changes in vertical ground reaction force during squat performed at various intensities of effort. In 2013, Zhang et al. [26] developed a force measuring system for determining the ground reaction force acting on foot during slow squat. The system permitted the evaluation of resultant force under the hindfoot and the forefoot. The study highlighted a greater weight distribution under the heel with respect to the forefoot, both for female and male subjects. Despite the innovative approach to overcome limitations of current instruments, the forces were measured as total resultant, without attention on single force component. In the present experiment, thanks to the use of the two force plates closed together, GRF components were evaluated. The major support of the hindfoot to the vertical component is confirmed, and a peak of 40% of the body weight was reached at the squatting pose, in contrast with the 10% BW peak at the forefoot. Moreover, the mediolateral component showed a different behavior for the two subareas. Indeed, the forefoot assumed a medial direction, while the hindfoot assumed a lateral position. These results appear substantially different to the total force obtained by the one-segment model that provides a considerable limitation and simplification in the analysis of foot placement and support. The examination and correlation of shear forces with the motion performance could be object of interest in activities that demand the foot rotation, as the take-off phase of the ski jumping.

In dynamics, due to the quasi static position and the limited motion, the intersegmental forces depicted constant values. The two-segment model gives the opportunity to appreciate the midtarsal contribution that reached a considerable value along the longitudinal axis (−2 N/kg). Only the sagittal plane analysis was reported for the dynamic evaluation of net joint moments and powers, because of the imperceptible contribution along other directions. As supposed, the ankle dynamics reveals similar results between the two models, without statistical significance for peak moments (*p* value = 0.35), peak power absorption (*p* value = 0.9), and peak power generation (*p* value = 0.75).

## 5. Conclusion

The central goal of this research discusses the application of a two-segment model for the biomechanical analysis of a partial body weight squat, with the purpose of investigating the foot joints and foot position with more details. The results of this study underline the important role of the foot during the squat practice and the possibility to achieve more accurate kinematic and dynamic information on the foot. The comparison of ankle results obtained with the simultaneous use of two-segment and one-segment models permitted to verify the accuracy of the method.

Kinematics and dynamics at the midtarsal joint reveal the fundamental role of the midfoot and the necessity to consider the separation of the forefoot from the hindfoot. The 3D kinematic analysis confirmed the limits of a 2D analysis and the complexity of the movement in the space. The coronal plane pointed out the different positions of the forefoot between subjects.

GRF estimation offers some useful information about foot poses, weight distribution between the forefoot and hindfoot, and squat strategies during the movement. The consideration of the magnitude and the direction of force components under different foot subareas could help the understanding of mechanical behaviors and body balance. As demonstrated by the comparison with the one total force registered, a similar interpretation seems to be impossible with the simplification of the foot as a one rigid segment. Not only the vertical component, but also the shear forces have reached values that should be considered during the squat analysis. The more detailed information might be applied in several investigations, and next researches could interrogate the correlation of GRF components with the knee and ankle joint positions.

Future works could be directed to a larger population for a complete standardization of the model. The analysis of different squat techniques could underline the interactions between variables. With a major squat depth or the addition of a final jump, it could be interesting to analyze the correlation of the forefoot position with the knee biomechanics with the attempt to evaluate how the strategy could influence the other joints. As declared by Schoenfeld [22], feet should be positioned in a comfortable stance that allows the knees to move in line with the toes. It also might be useful to verify the absence of statistical significance and overestimations between the two models in case of larger powers and net ankle moments exchanged, for example during the sprinting or jumping [34, 35]. The usage of the method is also interesting in the clinical fields, for example, with the attempt of a better comprehension of foot contribution during rehabilitation [46], to assess aged or pathological people motor capacities or to evaluate pharmacological interventions [47].

## Figures and Tables

**Table 1:** Marker configuration description and representation.

Name	Position	Description	Marker protocol
KNEE	Lateral femoral condyle	Apex of lateral femoral epicondyle	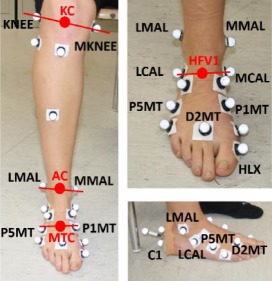
MKNEE	Medial femoral condyle	Apex of medial femoral epicondyle	
LMAL	Lateral malleolus	Apex of lateral malleolus	
MMAL	Medial malleolus	Apex of medial malleolus	
C1	Calcaneus 1	Apex of calcaneal tuberosity	
LCAL	Peroneal tubercle	Lateral calcaneus	
MCAL	Sustentaculumtali	Medial calcaneus	
P1MT	Metatarsal 1	Base of the 1st metatarsal	
P5MT	Metatarsal 5	Base of the 5th metatarsal	
D2MT	Head 2	Between 2nd and 3rd metatarsal head	
HLX	Hallux	Base of hallux	
Virtual landmarks			
KC	Knee joint center	Midpoint between MKNEE and KNEE	
AC	Ankle joint center	Midpoint between MMAL and LMAL	
HFV1	HFV1	Midpoint between MCAL and LCAL	
MTC	Midtarsal joint center	Midpoint between P1MT and P5MT	

**Table 2:** Segment local reference frames.

	Origin	*x*-axis	*y*-axis	*z*-axis	Graphic description
Shank	Ankle joint center AC	Cross product between *y*- and *z*-axes	Perpendicular to the plane identified by MMAL, LMAL, KC	Vector from AC to KC	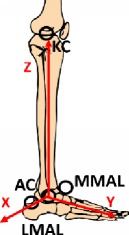
Foot	C1	Perpendicular to *y* axis and the vectors KC-AC	Vector from C1 to D2MT	Cross product between *x*- and *y*-axes	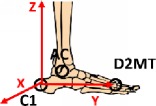
Hindfoot	C1	Cross product between vector C1-AC and vector C1-HFV1	Vector from C1 to HFV1	Cross product between *x*- and *y*-axes	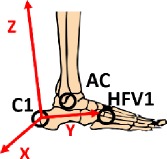
Forefoot	MTC	Cross product between *y*- and *z*-axes	Vector from MTC to D2MT	Perpendicular to the plane identified by P1MT, P5MT, and MTC